# Differential effects of formaldehyde exposure on airway inflammation and bronchial hyperresponsiveness in BALB/c and C57BL/6 mice

**DOI:** 10.1371/journal.pone.0179231

**Published:** 2017-06-07

**Authors:** Luanluan Li, Li Hua, Yafang He, Yixiao Bao

**Affiliations:** Department of Pediatric Pulmonology, Xinhua Hospital affiliated with Shanghai Jiao Tong University School of Medicine, Shanghai, China; Telethon Institute for Child Health Research, AUSTRALIA

## Abstract

Epidemiological evidence suggests that formaldehyde (FA) exposure may influence the prevalence and severity of allergic asthma. However, the role of genetic background in FA-induced asthma-like responses is poorly understood. In the present study, we investigated the nature and severity of asthma-like responses triggered by exposure to different doses of FA together with or without ovalbumin (OVA) in two genetically different mouse strains—BALB/c and C57BL/6. Both mouse strains were divided into two main groups: the non-sensitized group and the OVA-sensitized group. All the groups were exposed to 0, 0.5 or 3.0 mg/m^3^ FA for 6 h/day over 25 consecutive days. At 24 h after the final FA exposure, the pulmonary parameters were evaluated. We found that FA exposure induced Th2-type allergic responses in non-sensitized BALB/c and C57BL/6 mice. In addition, FA-induced allergic responses were significantly more prominent in BALB/c mice than in C57BL/6 mice. In sensitized BALB/c mice, however, FA exposure suppressed the development of OVA-induced allergic responses. Exposure to 3.0 mg/m^3^ FA in sensitized C57BL/6 mice also led to suppressed allergic responses, whereas exposure to 0.5 mg/m^3^ FA resulted in exacerbated allergic responses to OVA. Our findings suggest that FA exposure can induce differential airway inflammation and bronchial hyperresponsiveness in BALB/c and C57BL/6 mice.

## Introduction

Asthma is a chronic allergic disease characterized by airway inflammation and bronchial hyperresponsiveness. Over the last several decades, the prevalence of asthma has dramatically increased among children in both developed and developing countries [1, 2]. This rapid increase in prevalence is mainly due to exposure to environmental factors, including various indoor pollutants such as formaldehyde (FA) [3, 4].

FA is a common indoor air pollutant that is ubiquitously found in homes and buildings. The major sources of indoor FA are artificial boards made with formaldehyde-based resins, including plywood, blockboard, particleboard and medium-density fiberboard [5]. FA is also found in many household products such as textiles, cosmetics, plastics, and so on [6]. The possible effects of FA exposure on the prevalence and severity of asthma have been investigated in epidemiological studies [7–9]. Rumchev et al. found that indoor formaldehyde exposure was associated with an increased risk of asthma. Children exposed to formaldehyde levels ≥60μg/m^3^ were at increased risk of developing asthma [7]. In addition, Casset et al. found that exposure to formaldehyde in the home was sufficient to provoke sensitization and aggravate allergic symptoms in patients with asthma [8]. However, a study by Ezratty et al. reported that FA exposure had no significant deleterious effect on airway allergen responsiveness of patients with intermittent asthma; conversely, its exposure showed a trend toward a protective effect [9].

Several animal studies found that long-term exposure to FA could enhance bronchial responsiveness in rodents [10, 11]. In addition, FA exposure of mice sensitized with a house dust mite allergen was shown to aggravate eosinophilic airway inflammation [12]. Conversely, some recent studies have indicated that FA exposure could prevent the development of allergen-induced immune responses in rodents [13, 14], as indicated by the reduced inflammatory cell infiltration [13] and pro-inflammatory cytokine production [14].

The reasons for the conflicting reports in human and animal studies are unclear. However, differences in genetic background may be considered. It has been reported that genetic background can affect the degree of airway inflammation and bronchial responsiveness in rodent models of allergen-induced asthma [15, 16]. However, the role of the genetic background in asthma-like responses induced by FA exposure is poorly understood. In the present study, we used two genetically different mouse strains—BALB/c and C57BL/6—to investigate the effects of FA exposure on the development or exacerbation of allergic asthma.

## Materials and methods

### Animals

Male BALB/c and C57BL/6 mice (6–8 weeks old; 20–22 g) were purchased from the Hubei Province Experimental Animal Center (Wuhan, China). All mice were housed under specific pathogen-free conditions of 20–25°C and 50–70% relative humidity for one week before use. Food and water were available ad libitum for the duration of the experiments. This study was approved by the Animal Ethics Committee of Shanghai Jiao Tong University School of Medicine (Shanghai, China).

### Experimental groups

**Fig 1:**
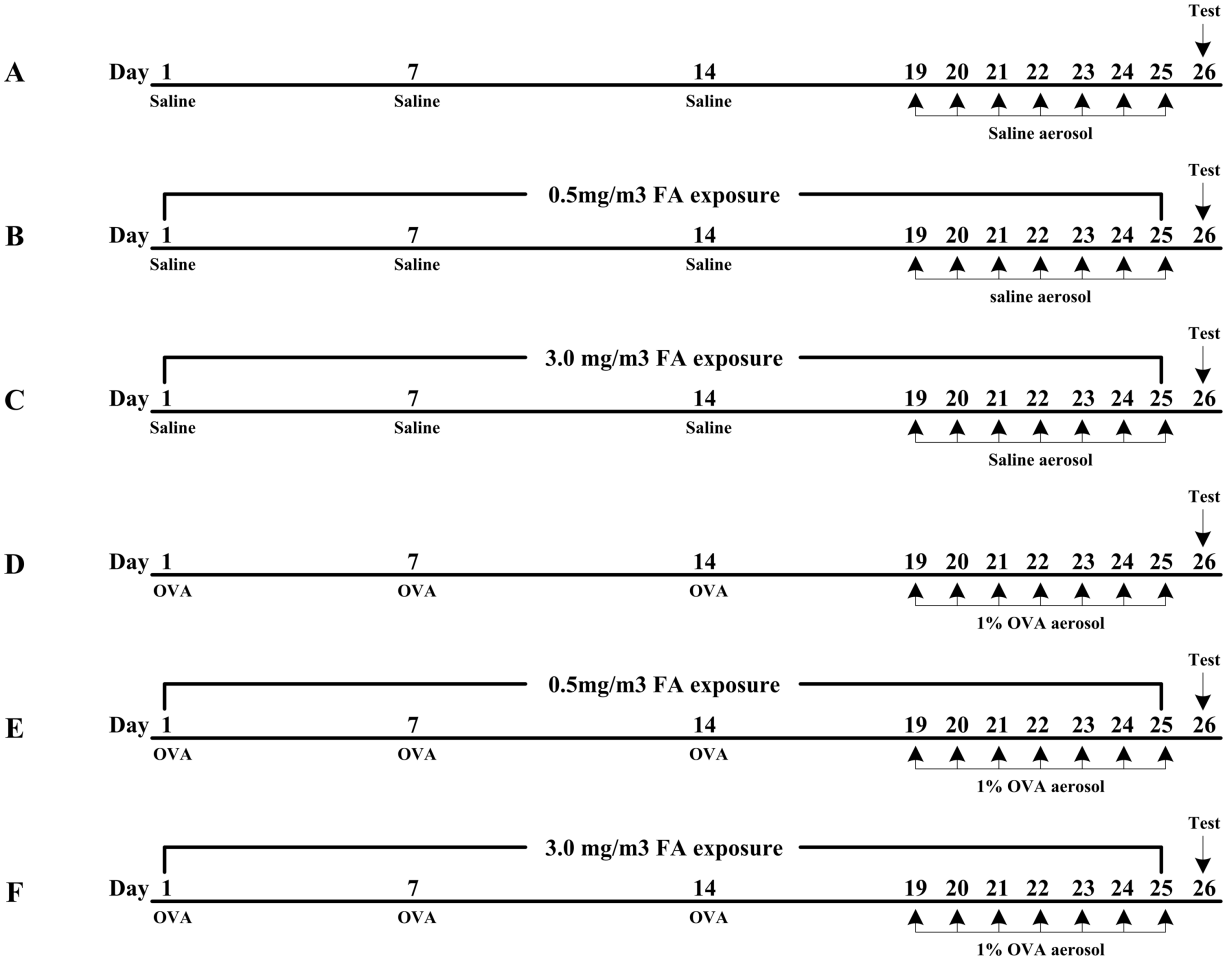
Experimental protocols for exposure of BALB/c and C57BL/6 mice to different doses of FA with or without OVA sensitization. In the sensitized group (D, E and F), mice were intraperitoneally injected with 50 μg OVA plus 1.75 mg aluminum hydroxide on days 1, 7 and 14 during the exposure period. The 1% aerosolized OVA challenge was carried out on days 19–25. In the non-sensitized group (A, B and C), mice were sham sensitized by 0.9% saline injections and challenged with 0.9% saline aerosols following the same protocols. Both the non-sensitized and sensitized mice were exposed to three concentrations of FA (0, 0.5 and 3.0 mg/m^3^) for 6 h/day over 25 consecutive days. All data shown are representative of two separate experiments.

Both BALB/c and C57BL/6 mice were randomly divided into two main groups: the non-sensitized group and the sensitized group. Based on the exposure concentration of FA, each group was then divided into three subgroups (n = 14): the naïve group (non-sensitized and no FA exposure), the 0.5 FA group (non-sensitized and 0.5 mg/m^3^ FA), the 3.0 FA group (non-sensitized and 3.0 mg/m^3^ FA), the OVA group (sensitized and no FA exposure), the 0.5 FA + OVA group (sensitized and 0.5 mg/m^3^ FA), and the 3.0 FA + OVA group (sensitized and 3.0 mg/m^3^ FA). At 24 h after the final FA exposure, 8 mice from each subgroup were assessed for airway inflammation, pulmonary cytokine production and serum OVA-specific immunoglobulin. The remaining 6 mice in each subgroup were used to assess bronchial responsiveness. The experimental protocols in this study are shown in Fig 1.

### Sensitization protocols

Mice in the sensitized group were intraperitoneally injected with 50μg OVA plus 1.75 mg aluminum hydroxide in 0.3 ml 0.9% saline on days 1, 7 and 14. The aerosol challenge was carried out by ultrasonic nebulization with 1% OVA for 30 min on days 19–25. Mice in the non-sensitized group were sham sensitized by 0.9% saline injections and challenged with 0.9% saline aerosols following the same protocols.

### Formaldehyde exposure

Gaseous FA was generated from a 10% formalin solution (Sigma, St. Louis, MO, USA) using a standard gas generator (Wuhan, China) and delivered to two 20-L glass exposure chambers. The parameters of the FA gas generator were conditioned at a temperature of 23±0.5°C, a relative humidity of 45±5% and an airflow rate of 3.0 L/min. The desired gaseous FA concentrations could be achieved by adjusting the ratio of the formalin solution to distilled water. The FA concentrations in the chambers were monitored every two hours during exposure using a Formaldemeter (4160–2, Interscan, Simi Valley, CA, USA). The choice of FA exposure concentrations in our experiment was based on recent (0.5 mg/m^3^) and previous (3.0 mg/m^3^) occupational exposure limits for formaldehyde in China. All groups of mice were exposed to 0, 0.5 or 3.0 mg/m^3^ FA for 6 h/day over 25 consecutive days.

### Measurement of OVA-specific immunoglobulin

At 24 h after the final FA exposure, mice were deeply anesthetized via intraperitoneal injection of sodium pentobarbital (100 mg/kg). Whole blood was collected by cardiac puncture and then allowed to coagulate at room temperature for ≥30 min. Serum was obtained by centrifugation at 3000 rpm for 15 min. The concentrations of OVA-specific IgE, IgG1 and IgG2a in the sera were measured with enzyme-linked immunosorbent assay (ELISA) kits (BlueGene Biotech, Shanghai, China) according to the manufacturer’s instructions.

### Assessment of pulmonary cytokine production and airway inflammation

After drawing the blood, bronchoalveolar lavage was performed twice with 1 ml of ice-cold saline using a tracheal cannula. The bronchoalveolar lavage fluid (BALF) was collected and centrifuged at 4°C, 3000 rpm for 10 min. The supernatant was separated and stored at -80°C for cytokine analysis. The concentrations of IL-4, IL-5, IL-13 and IFN-γ in the BALF were measured using ELISA kits (eBioscience, San Diego, CA, USA). Lower detection limits were 4 pg/ml for IL-4, 4 pg/ml for IL-5, 4 pg/ml for IL-13 and 15 pg/ml for IFN-γ.

The severity of airway inflammation was evaluated by counting the cells in the BALF. The centrifuged deposit was resuspended in 0.5 ml of 0.9% saline to determine the total and differential cell counts. The numbers of total cells, eosinophils, lymphocytes and neutrophils in the BALF were counted using an automated hematology analyzer (MTN-21, Motenu, China).

### Assessment of bronchial responsiveness

At 24 h after the final FA exposure, bronchial responsiveness to methacholine (MCh) was measured using an AniRes 2005 lung function system (Bestlab 2.0, Beijing, China). The trachea was cannulated under anesthesia with an intraperitoneal injection of 100 mg/kg sodium pentobarbital and then connected to a computer-controlled ventilator. The respiratory rate was preset at 95/min, and the time ratio of expiration to inspiration was preset at 1.5:1. MCh at doses of 0.025, 0.05, 0.1, and 0.2 mg/kg was administered at 5-min intervals through an injector needle placed into the jugular vein. After each MCh injection, the inspiratory resistance and expiratory resistance were recorded to determine the bronchial responsiveness to increasing doses of MCh.

### Statistical analysis

All data were expressed as the mean ± SE. Differences between groups were analyzed using the Student’s t test and the analysis of variance (ANOVA) followed by the Bonferroni post-test. Statistical analyses were performed using the GraphPad Prism 5.0 software package (Graph-Pad Software, San Diego, California, USA). A p value less than 0.05 was considered statistically significant.

## Results

### Effect of FA exposure on cytokine production in the BALF

**Fig 2:**
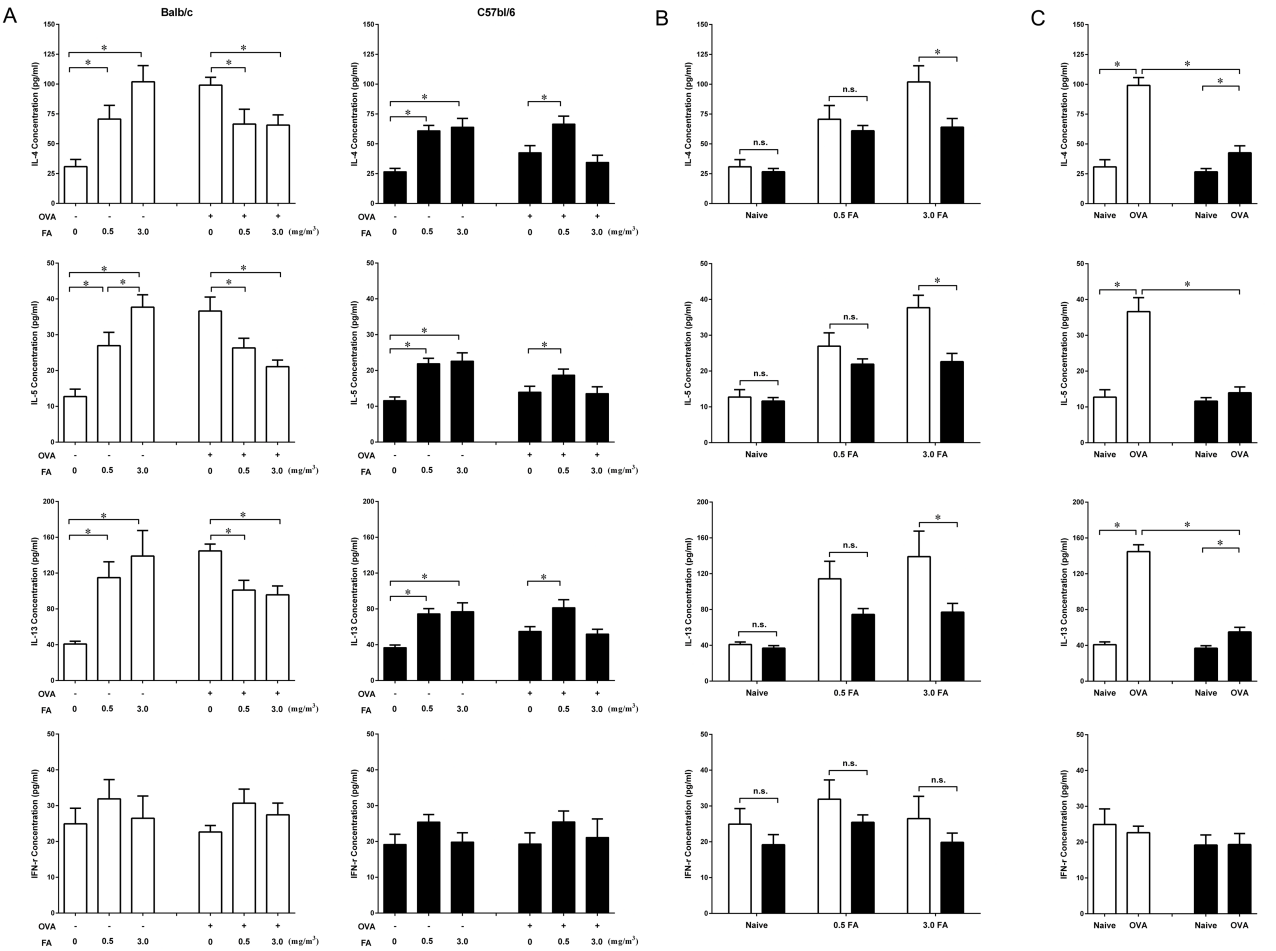
Effect of FA exposure on cytokine production in the BALF from BALB/c and C57BL/6 mice with or without OVA sensitization. At 24 h after the final FA exposure, the BALF was collected from all groups of mice, and the concentrations of IL-4, IL-5, IL-13 and IFN-γ were measured by ELISA. (A) Effect of FA exposure on cytokine production in the non-sensitized and sensitized BALB/c (□) and C57BL/6 (■) mice. (B) Comparison of the effect of inhaled FA dose on cytokine production between BALB/c (□) and C57BL/6 (■) mice. (C) Differences in cytokine production after sensitization with OVA in BALB/c (□) and C57BL/6 (■) mice. Data are presented as the mean± SE (n = 8 mice/group) (* p < 0.05).

In the non-sensitized group, exposure to 0.5 mg/m^3^ and 3.0 mg/m^3^ FA significantly increased the production of Th2 cytokines (IL-4, IL-5 and IL-13) in a dose-dependent manner in both BALB/c and C57BL/6 mice (Fig 2A). However, BALB/c mice exposed to 3.0 mg/m^3^ FA had much higher concentrations of IL-4, IL-5 and IL-13 in the BALF than that of C57BL/6 mice (Fig 2B). Both mouse strains showed no significant difference in Th1 cytokine (IFN-γ) production among the various FA doses.

Sensitization with OVA in BALB/c mice dramatically increased Th2 cytokine production in the BALF (Fig 2C), but simultaneous exposure to 0.5 mg/m^3^ and 3.0 mg/m^3^ FA during sensitization markedly decreased the Th2 cytokine levels (Fig 2A). In sensitized C57BL/6 mice, exposure to 3.0 mg/m^3^ FA during OVA sensitization also decreased the production of Th2 cytokines, but this decrease was not statistically significant. Unlike the corresponding BALB/c mice, sensitized C57BL/6 mice exposed to 0.5 mg/m^3^ FA presented significantly increased concentrations of IL-4, IL-5 and IL-13 in the BALF compared with sensitized C57BL/6 mice without FA exposure. There were no significant differences in the concentrations of IFN-γ regardless of FA exposure in the sensitized BALB/c and C57BL/6 mice.

Sensitization with OVA resulted in markedly increased Th2 cytokine production such as IL-4 and IL-13 in the BALF of both BALB/c and C57BL/6 mice (Fig 2C). However, the concentrations of Th2 cytokines after OVA sensitization were significantly higher in BALB/c mice than in C57BL/6 mice, suggesting that BALB/c mice exhibited a more Th2-like response to OVA than C57BL/6 mice.

### Effect of FA exposure on airway inflammation

**Fig 3:**
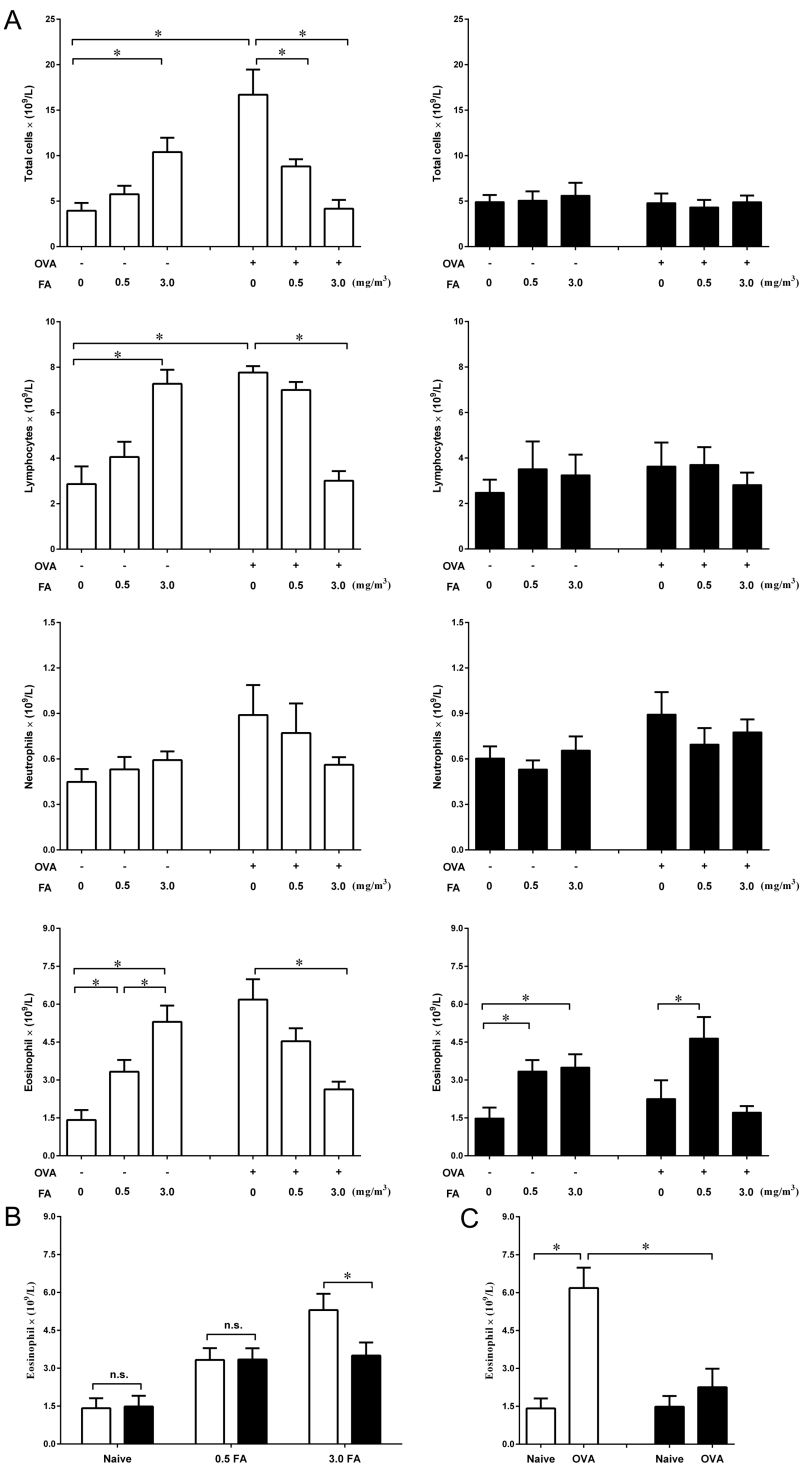
Effect of FA exposure on airway inflammation in BALB/c and C57BL/6 mice with or without OVA sensitization. Airway inflammation was assessed using inflammatory cell counts in the BALF. (A) Effect of FA exposure on inflammatory cell counts in the non-sensitized and sensitized BALB/c (□) and C57BL/6 (■) mice. (B) Comparison of the effect of inhaled FA dose on eosinophil counts between BALB/c (□) and C57BL/6 (■) mice. (C) Differences in eosinophil counts after sensitization with OVA in BALB/c (□) and C57BL/6 (■) mice. Data are presented as the mean± SE (n = 8 mice/group) (* p < 0.05).

To examine the effect of FA exposure on airway inflammation, total and differential cell counts in the BALF were determined (Fig 3A). In the non-sensitized BALB/c and C57BL/6 mice, exposure to 0.5 mg/m^3^ or 3.0 mg/m^3^ FA induced a significant increase in eosinophil counts compared with the respective naïve mice (Fig 3A). Nevertheless, the number of eosinophils after exposure to 3.0 mg/m^3^ FA was much higher in BALB/c mice than in C57BL/6 mice (Fig 3B). BALB/c mice exposed to 3.0 mg/m^3^ FA also exhibited an increase in the number of total cells and lymphocytes (Fig 3A).

BALB/c mice sensitized with OVA presented eosinophilic airway inflammation (Fig 3C), but simultaneous exposure to 3.0 mg/m^3^ FA during sensitization significantly reduced OVA-induced eosinophilic inflammation (Fig 3A). In sensitized C57BL/6 mice, however, exposure to 0.5 mg/m^3^ FA during sensitization led to a significant increase in the number of eosinophils in the airway. In addition, BALB/c mice exposed to 3.0 mg/m^3^ FA during sensitization exhibited a marked decrease in the number of total cells and lymphocytes.

In C57BL/6 mice, OVA sensitization did not induce a significant increase in the number of eosinophils (Fig 3C), which was consistent with the low concentration of IL-5 in the BALF (Fig 2C). However, the concentrations of IL-4 and IL-13 in the BALF were significantly increased in C57BL/6 mice after OVA sensitization (Fig 2C). These findings suggest that sensitization with OVA may induce a relatively weak Th2-like response in C57BL/6 mice. In addition, the number of eosinophils in the BALF after OVA sensitization was much higher in BALB/c mice than in C57BL/6 mice, supporting that BALB/c mice were more prone to Th2-like response than C57BL/6 mice.

### Effect of FA exposure on bronchial hyperresponsiveness

**Fig 4:**
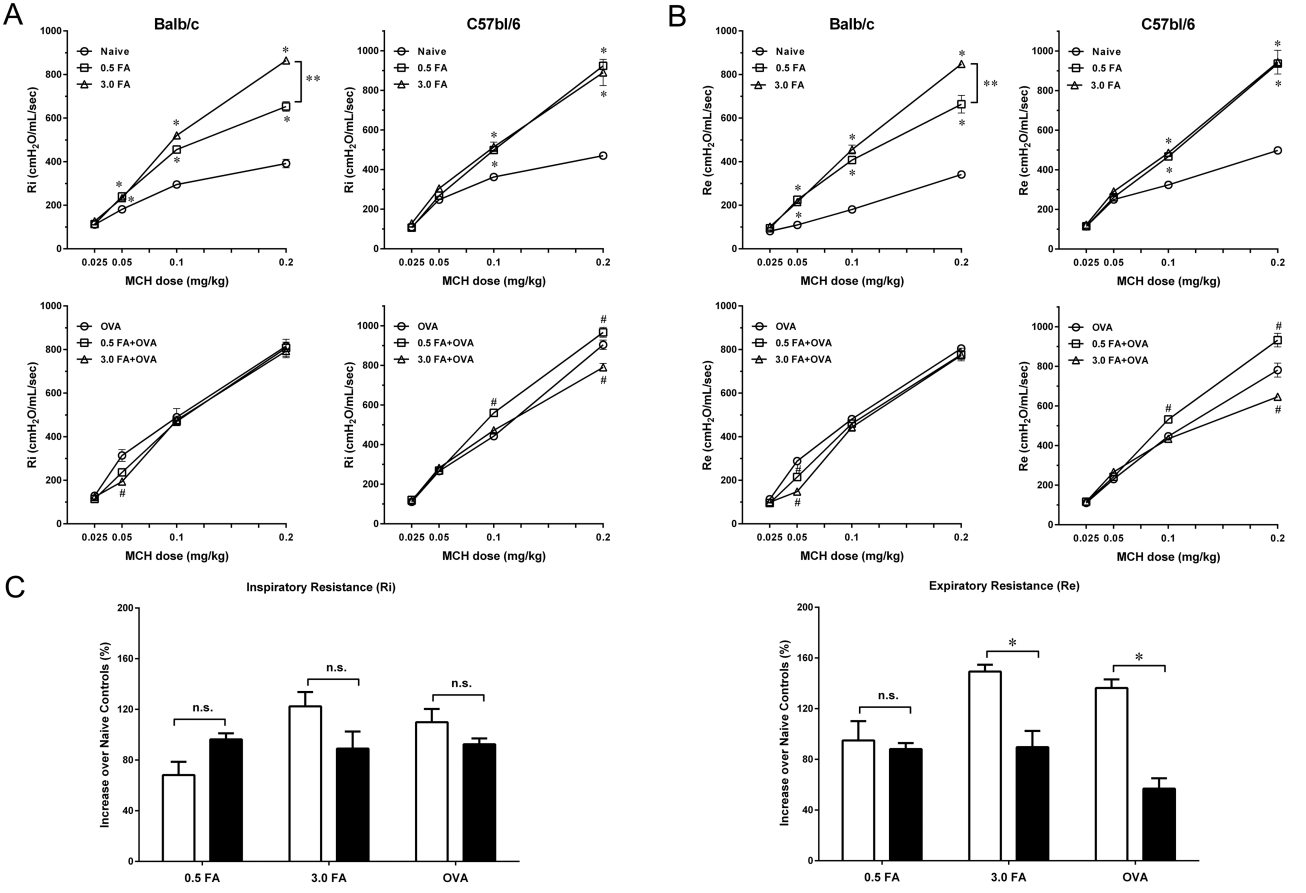
Effect of FA exposure on bronchial responsiveness to methacholine in BALB/c and C57BL/6 mice with or without OVA sensitization. Inspiratory resistance (Ri) and expiratory resistance (Re) were recorded to determine the bronchial responsiveness to methacholine. (A) Effect of inhaled FA dose on inspiratory resistance (Ri) in the non-sensitized and sensitized BALB/c and C57BL/6 mice. (B) Effect of inhaled FA dose on expiratory resistance (Re) in the non-sensitized and sensitized BALB/c and C57BL/6 mice. Data are presented as the mean± SE (n = 6 mice/group). * p < 0.05 compared with the naïve group. **#** p < 0.05 compared with the OVA group. ** p < 0.05 for the 0.5 FA group compared with the 3.0 FA group. (C) Comparison of the increases of Ri and Re after FA exposure between BALB/c and C57BL/6 mice. Data are expressed as % maximal increases over naïve controls (mean± SE) (* p < 0.05).

In the non-sensitized group, exposure to 0.5 mg/m^3^ or 3.0 mg/m^3^ FA induced bronchial hyperresponsiveness in both BALB/c and C57BL/6 mice (Fig 4A and 4B). BALB/c mice exposed to 3.0 mg/m^3^ FA exhibited a significant increase in airway response to MCh at doses of 0.1 and 0.2 mg/kg compared with BALB/c mice exposed to 0.5 mg/m^3^ FA. In C57BL/6 mice, however, no significant difference in airway resistance was observed between mice exposed to 0.5 mg/m^3^ and 3.0 mg/m^3^ FA at any dose of MCh. In addition, exposure of BALB/c mice to 3.0 mg/m^3^ FA induced a markedly increased expiratory resistance (increase over naïve controls) that was much greater than the increase in C57BL/6 mice (Fig 4C). There were no significant differences in the increases of inspiratory resistance among the various FA doses between two mouse strains.

In the sensitized group, airway response to MCh (0.05 mg/kg) in sensitized BALB/c mice exposed to 3.0 mg/m^3^ FA was significantly decreased compared with sensitized BALB/c mice without FA exposure (Fig 4A and 4B). Airway response to MCh (0.2 mg/kg) was also decreased in sensitized C57BL/6 mice after exposure to 3.0 mg/m^3^ FA. However, sensitized C57BL/6 mice exposed to 0.5 mg/m^3^ FA exhibited a significant increase in airway resistance when administered MCh at doses of 0.1 and 0.2 mg/kg. In addition, BALB/c mice sensitized with OVA displayed a significant increase in expiratory resistance compared with C57BL/6 sensitized with the same allergen (Fig 4C).

### Effect of FA exposure on OVA-specific immunoglobulin in the sera

**Fig 5:**
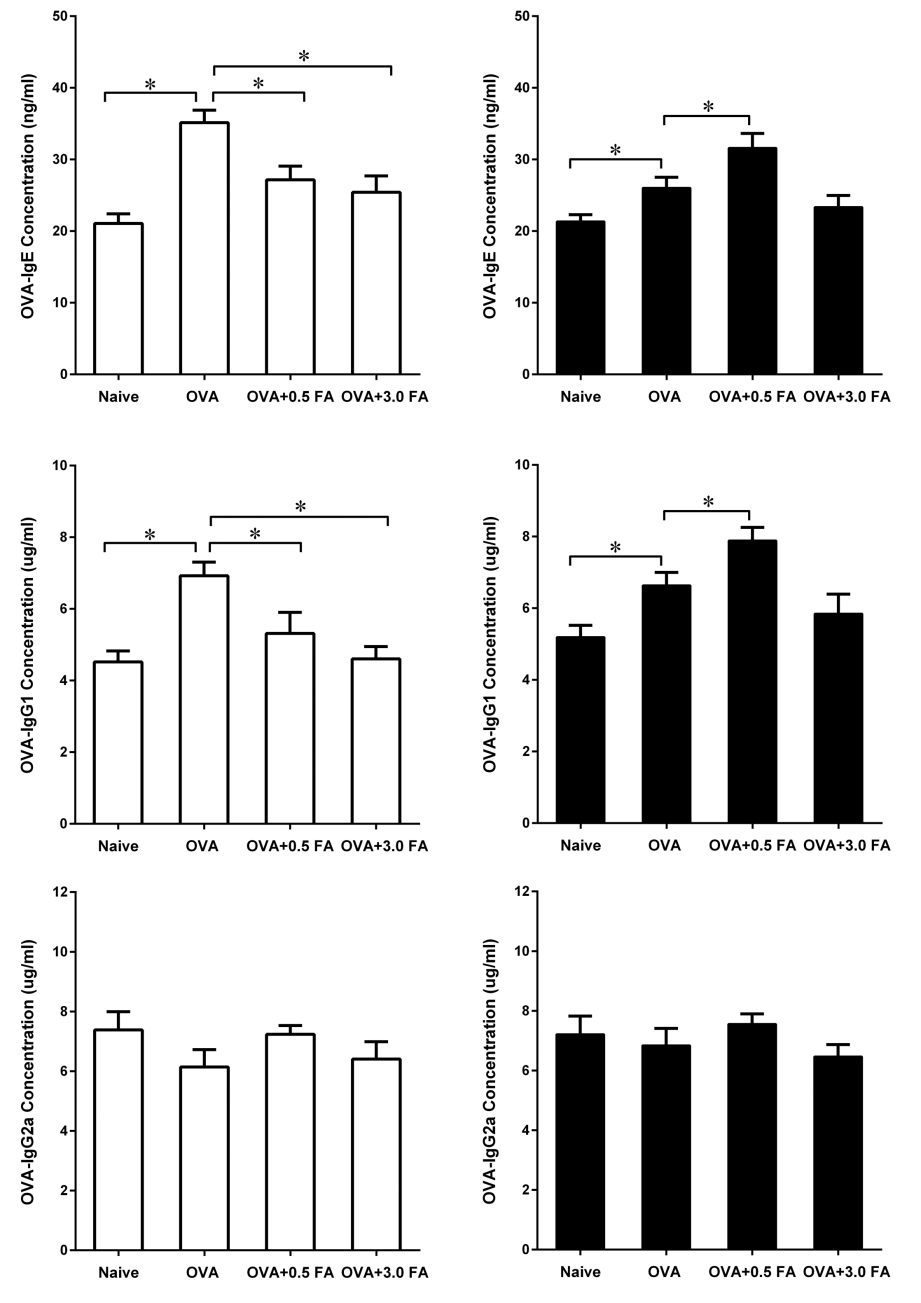
Effect of FA exposure on serum OVA-specific immunoglobulin in BALB/c and C57BL/6 mice. At 24 h after the final FA exposure, blood samples were collected from BALB/c (□) and C57BL/6 (■) mice, the concentrations of OVA-specific IgE, IgG1 and IgG2a in the sera were measured by ELISA. Data are presented as the mean± SE (n = 8 mice/group) (* p < 0.05).

Sensitization with OVA significantly increased the production of serum OVA-IgE and OVA-IgG1 in both mouse strains when compared with naïve controls (Fig 5). However, exposure to 0.5 mg/m^3^ or 3.0 mg/m^3^ FA in sensitized BALB/c mice led to a significant decrease in serum OVA-IgE and OVA-IgG1 production. In contrast, sensitized C57BL/6 mice exposed to 0.5 mg/m^3^ FA exhibited a significant increase in serum OVA-IgE and OVA-IgG1 production compared with sensitized C57BL/6 mice without FA exposure. In addition, exposure to various concentrations of FA did not affect the production of OVA-IgG2a in sensitized BALB/c and C57BL/6 mice.

## Discussion

In this study, we investigated the effects of different doses of FA exposure on the development and exacerbation of allergic responses in two genetically different mouse strains—BALB/c and C57BL/6. The results indicated that exposure to FA induced airway inflammation and bronchial hyperresponsiveness in non-sensitized BALB/c and C57BL/6 mice. In addition, FA-induced allergic responses were significantly more prominent in BALB/c mice than in C57BL/6 mice. However, simultaneous exposure to FA during sensitization led to suppressed OVA-induced allergic responses in both strains of mice. The exception was C57BL/6 mice that were exposed to 0.5 mg/m^3^ FA during OVA sensitization; these mice exhibited enhanced allergic responses to OVA after FA exposure.

FA is a common indoor air pollutant, and its irritant effects on the airway have been reported [17–19]. In the present study, we found that FA exposure significantly increased the production of Th2 cytokines, including IL-4, IL-5 and IL-13, in the BALF of non-sensitized BALB/c and C57BL/6 mice. The production of Th1 cytokines such as IFN-γ was not affected by exposure to different concentrations of FA. We therefore propose that FA exposure can promote Th2-type inflammatory responses in both BALB/c and C57BL/6 mice, which is consistent with increased eosinophil infiltration in the BALF of both mouse strains after FA exposure. The exact role of FA in airway inflammation is still unclear. It is possible that FA may induce airway inflammation via various signaling pathways such as the MAPK and NF-κb pathways [20]. Moreover, FA exposure has been reported to increase the production of intracellular reactive oxygen species (ROS) [21]. It is plausible that FA may enhance Th2 cytokine production and eosinophil infiltration by regulating ROS generation. Nevertheless, we also found that FA-induced airway inflammation was significantly more prominent in BALB/c mice than in C57BL/6 mice. It is likely that BALB/c mice are more susceptible to FA-induced inflammatory responses than C57BL/6 mice. The genetic differences between these two mouse strains may be responsible for high or low airway responses to FA exposure, which can reflect the response state in atopic or non-atopic individuals, respectively.

The adverse effects of FA exposure on pulmonary function have been reported in individuals exposed to FA. Kriebel et al. found that among students in the anatomy dissection laboratory, exposure to 1.1 ppm formaldehyde for 2.5 h/wk reduced the peak expiratory flow (PEF) by 1% per ppm [22]. Neghab et al. also observed that long-term occupational exposure to formaldehyde was associated with impaired lung function, as evidenced by decreased vital capacity (VC), forced vital capacity (FVC) and forced expiratory volume in the first second (FEV_1_) [23]. Consistent with these human studies, the results of our animal studies indicated that FA exposure significantly increased the airway resistance during inspiration and expiration in non-sensitized BALB/c and C57BL/6 mice. It is likely that formaldehyde may contribute to the regulation of S-nitrosoglutathione (GSNO) metabolism and thus influence smooth muscle tone in the airways [24]. GSNO is considered an endogenous bronchodilator involved in airway smooth muscle relaxation [25]. The catabolism of GSNO depends on S-nitrosoglutathione reductase (GSNOR), an enzyme responsible for the endogenous and exogenous clearance of FA [26, 27]. Therefore, we propose that upon FA exposure, GSNOR is activated to metabolize FA, thus leading to an increased GSNO breakdown, which contributes to the FA-induced airway hyperresponsiveness to MCh. In addition, we found that FA exposure induced more pronounced bronchial hyperresponsiveness in BALB/c mice than in C57BL/6 mice, especially in terms of expiratory resistance. There were no significant differences in inspiratory resistance between two mouse strains after FA exposure. The differences in expiratory resistance between two mouse strains may be related to the degree of airway obstruction. Physiologically, airflow resistance due to airway obstruction would be elevated during expiration compared with that during inspiration. Therefore, we speculate that exposure of BALB/c mice to FA may lead to more severe airway obstruction than that of C57BL/6 mice, thus resulting in differences in expiratory resistance between two mouse strains.

Aside from the allergic effects of FA exposure in non-sensitized mice, the most interesting findings were that exposure to 0.5 mg/m^3^ or 3.0 mg/m^3^ FA in sensitized BALB/c mice suppressed the development of OVA-induced allergic responses. Exposure to 3.0 mg/m^3^ FA during sensitization also led to the suppression of allergic reactions in sensitized C57BL/6 mice. A possible reason for this is that long-term exposure to FA during sensitization may induce the development of immune tolerance, which reduces allergic responses to antigen sensitization and challenges [28]. Another possible explanation is that repeated FA inhalation may modify the synthesis of anti-OVA immunoglobulin [29], which in turn impairs the activation of mast cells and consequently leads to the suppression of OVA-induced allergic responses. This was confirmed by the reduced OVA-specific IgE and IgG1 production observed in both sensitized mouse strains exposed to FA in our study. In contrast with 3.0 mg/m^3^ FA, inhalation of 0.5 mg/m^3^ FA in sensitized C57BL/6 mice resulted in exacerbated OVA-induced allergic responses. The paradoxical effects of FA in sensitized C57BL/6 mice might be explained by the differences in exposure levels. We propose that exposure to a low dose of FA (0.5 mg/m^3^) may enhance sensitization to the OVA antigen in C57BL/6 mice, whereas exposure to higher doses of FA (3.0 mg/m^3^) may reduce the allergic responses to the OVA antigen. Our results were in partial agreement with previous studies, which found that exposure to a low dose of FA (0.5% or 0.25 ppm) could enhance allergen-induced eosinophilic airway inflammation or bronchial hyperresponsiveness in rodents [12, 30].

Surprisingly, we found that exposure to 0.5 mg/m^3^ FA during sensitization had different effects in BALB/c and C57BL/6 mice regarding OVA-induced allergic responses. It was also found that BALB/c mice exhibited a more Th2-like response to the OVA antigen than C57BL/6 mice. Moreover, FA-induced allergic responses were more prominent in BALB/c mice than in C57BL/6 mice. Thus, it is possible that BALB/c mice are more sensitive to simultaneous exposure to FA together with the OVA antigen than C57BL/6 mice. Exposure to 0.5 mg/m^3^ FA in sensitized BALB/c mice was sufficient to suppress allergic responses to OVA. We speculate that the threshold of suppression for these two mouse strains may require distinct doses of FA, resulting in differential effects on OVA-induced allergic responses. This discrepancy may be due to differences in the genetic background of BALB/c and C57BL/6 mice. Our findings can help explain the conflicting results regarding the effects of FA exposure on asthma severity in human studies.

In conclusion, FA exposure can induce differential airway inflammation and bronchial hyperresponsiveness in BALB/c and C57BL/6 mice. The genetic background is an important factor that affects the role of FA exposure in the development and exacerbation of allergic responses, which should be considered in the design and interpretation of relevant studies.
